# Stability Prediction of 2H–MoO_2_ Monolayer as a Platform for Photonic Devices: from Thermodynamics to the Excitonic Effects through First-Principles Calculations

**DOI:** 10.1021/acsomega.5c10173

**Published:** 2026-01-30

**Authors:** Gleidson S. Costa, Celso Alves do Nascimento Júnior, Alexandre Silva Santos, Maurício Jeomar Piotrowski, Celso Ricardo Caldeira Rêgo, Diego Guedes-Sobrinho, Carlos Maciel O. Bastos, Luiz A. Ribeiro Júnior, Alexandre C. Dias

**Affiliations:** † Department of Mathematics, University of Brasília, Brasília 70919-970, Brazil; ‡ Department of Physics, Federal University of Ouro Preto, 35400-000 Ouro Preto, Brazil; § Institute of Physics, University of Brasília, Brasília 70919-970, Brazil; ∥ Optical Spectroscopy Laboratory, Institute of Physics, University of Brasília, Brasília 70919-970, Brazil; ⊥ Department of Physics, Federal University of Pelotas, P.O. Box 354, Pelotas 96010-900, Brazil; # 150232 Karlsruhe Institute of Technology (KIT), Institute of Nanotechnology, Hermann-von-Helmholtz-Platz, Eggenstein-Leopoldshafen 76344, Germany; ¶ Chemistry Department, Federal University of Paraná, CEP 81531 − 980, Curitiba 81531-980, Brazil; ∇ Institute of Physics and International Center of Physics, University of Brasília, Brasília 70919-970, Brazil; ○ Institute of Physics, University of Brasília, Brasília 70910-900, Brazil; ⧫ Computational Materials Laboratory, LCCMat, Institute of Physics, University of Brasília, Brasília 70910-900, Brazil

## Abstract

The conception, study, and development of two-dimensional (2D) materials have expanded the frontiers of next-generation optoelectronic devices. Representative of this class, the MoO_2_ monolayer in its 2H phase was investigated here with respect to its structural, electronic, optical, and excitonic properties, through the PBE level for structural and electronic properties, being the electronic band gap correct at the HSE06 level, the optical and excitonic properties were obtained by solving the Bethe-Salpeter equation. The structural stability was also investigated at the dynamical (phonons), thermodynamic (AIMD), and mechanical (elastic constants) levels, ensuring the stability of this monolayer at all levels. This 2D transition-metal dioxide exhibits semiconducting behavior with a HSE06 direct band gap of 2.50 eV, where spin–orbit coupling is weak. We also observe spin degeneracy breaking in the valence bands close to the Fermi level in the vicinity of the K and K′ valleys and along the connecting path between them. Excitonic band-structure analysis revealed a binding energy of 0.38 eV, which gives rise to significant excitonic effects in the linear optical response. The response is isotropic across the infrared and visible ranges, extending to the onset of the ultraviolet spectrum.

## Graphical abstract

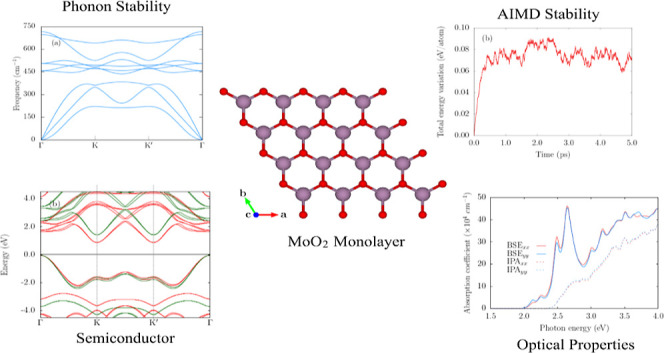

## 1 Introduction

Graphene has emerged as a cornerstone in the development of 2D materials, owing to its unprecedented properties arising from quantum confinement along the nonperiodic direction.^1^ Since its isolation in the early 2000s, this material has stimulated the rapid search for novel 2D systems, given their potential in high-frequency electronics and broadband optoelectronics. Beyond graphene, other 2D monolayers and their stackable counterparts, such as van der Waals (vdW) heterostructures,^2^ have advanced the state-of-the-art in materials science by demonstrating functionalities suitable for emerging and promising technologies.^3,4^

Among these, transition-metal dichalcogenides (TMDCs) have attracted considerable attention, since their graphene-like honeycomb lattice, often crystallizing in the 2H phase,^5^ is combined with semiconducting behavior, in contrast to graphene’s semimetallic nature. TMDCs adopt the stoichiometry MX_2_, where a transition-metal atom M is intercalated by two chalcogen atoms X. Several TMDCs, such as MoS_2_,^6^ WS_2_,^7^ MoSe_2_,^8^ MoTe_2_,^9^ WSe_2_,^10,11^ and CrS_2_,^12^ have been usually investigated for their uses in biosensors, optoelectronics, flexible electronics, photonics, energy storage, and photovoltaics. Thus, advancing within the broader family, transition-metal dioxides (TMDOs) have also become the focus of interest, given their structural, electronic, optical, and excitonic characteristics. Reported studies include NiO_2_,^13^ CrO_2_,^14^ ZrO_2_,^15,16^ HfO_2_,^15,17^ MnO_2_,^18^ PtO_2_,^19^ OsO_2_,^20^ and RuO_2_,^21,22^ as well as systematic works surveying this family.^23,24^ However, a satisfactory understanding of the optical properties of these materials considering excitonic effects is far from ideal.

In this work, we investigate the 2H–MoO_2_ monolayer, combining density functional theory (DFT) calculations with many-body methods. We assess thermodynamic, mechanical, electronic, and excitonic properties. Phonon dispersion, elastic constants calculations, and *ab initio* molecular dynamics (AIMD) confirm stability.^25^ Electronic properties are evaluated with plain semilocal and nonlocal hybrid exchange–correlation functionals, including spin–orbit coupling (SOC). The linear optical response is explored through the independent particle approximation (IPA) and the Bethe–Salpeter Equation (BSE) formalism, using maximally localized Wannier functions (MLWF-TB) as input for excitonic calculations.^26–29^

## 2 Theoretical Methodology and Computational Details

First-principles simulations were performed within the DFT framework using the Vienna *Ab Initio* Simulation Package (VASP).^30,31^ Structural and electronic properties were initially explored within the Perdew–Burke–Ernzerhof (PBE) functional, a widely used member of the generalized gradient approximation (GGA) family.^32,33^ As is well-known, PBE underestimates band gaps due to the derivative discontinuity and self-interaction errors,^34,35^ thereby, we also employed the screened hybrid HSE06 functional,^36,37^ which partially incorporates exact exchange, improving band gap accuracy with manageable computational cost.

Calculations were performed using the projector augmented-wave (PAW) method.^38,39^ Structural relaxation based on unit cell as depicted in Figure 1 was achieved with a plane-wave cutoff of 1050 eV, an energy convergence criterion of 10^–6^ eV, and interatomic forces convergence below 0.01 eV/°A was used to minimize atomic forces and optimize the stress tensor. A 16 × 16 × 1 Monkhorst–Pack mesh (corresponding to a **k**-point density of 40 A^–1^) ensured Brillouin-zone integration. For density-of-states (DOS) calculations, a denser 33 × 33 × 1 grid was used (corresponding to a **k**-point density of 80 A^–1^). Phonon dispersion and thermodynamic properties were computed with Phonopy^40^ using a 4 × 4 × 1 supercell, 24 × 24 × 1 **k**-mesh, and a vacuum of 17.5 Å along ẑ. In addition, thermodynamic properties were assessed with those same packages by computing the Helmholtz free energy, entropy, and heat capacity at constant volume. AIMD simulations employed the FHI-aims code^25^ (within light tier 1 basis set) with a Nose–Hoover thermostat at 300 K (thermalization), a time step of 1 fs, and a total simulation time of 5 ps for a 4 × 4 × 1 supercell with a 24 × 24 × 1 **k**-mesh.

**Figure 1:**
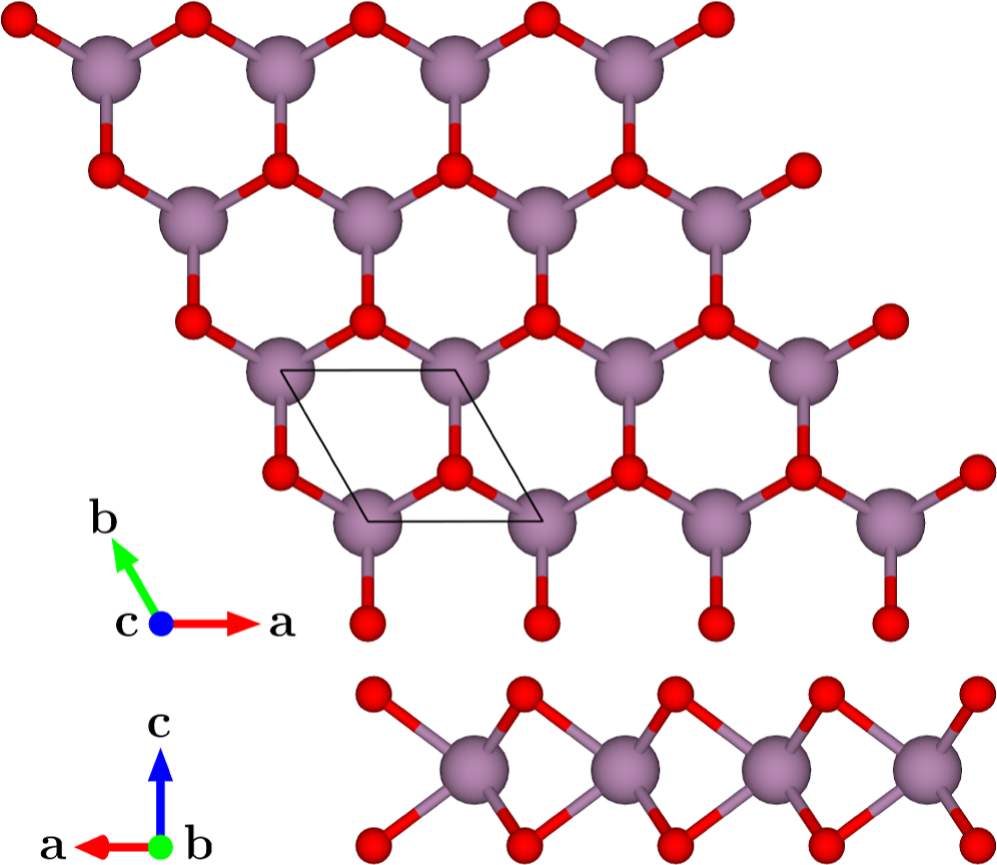
Top and side views of the 2H–MoO_2_ monolayer crystal structure with the unit cell lines highlighted, where Mo atoms are shown in purple and O atoms in red.

Elastic constants were extracted via the stress–strain method and analyzed through Hooke’s law. For the hexagonal lattice, only *C*_11_ and *C*_12_ are independent, leading to isotropic expressions for the shear modulus *G*, Young’s modulus *Y*, and Poisson’s ratio ν.^41–44^ For optical properties, we solved the BSE with WanTiBEXOS,^28^ using MLWF-TB Hamiltonians constructed from HSE06+SOC band structures via VASP and Wannier90,^26^ considering *d*- and p-orbital projections for Mo and O atomic species, respectively. A 2D truncated Coulomb potential (V2DT)^45^ was adopted with eight conduction and two valence bands over a 49 × 49 × 1 **k**-grid and a Gaussian smearing value of 0.05 eV (for a more accurate description of the dielectric constants).

More specifically, through the generalized Hooke’s law, the elastic constants relate to the stress response **σ** to externally applied strain **ϵ** can be given by^46^ the following expression

1 $$\sigma_{i}=\sum_{1}^{6}C_{ij}\epsilon_{j}$$

where the coefficients *C*_*ij*_ constitute the so-called elastic stiffness tensor **C**, which can be reduced to a lower order by exploiting physical symmetries present in the structure. In our case, given the MoO_2_ hexagonal unit cell, **C** is further simplified by presenting a small number of independent elements and taking the form

2 $$C=\left(\begin{array}{lll} C_{11} & C_{12} & 0\\ C_{12} & C_{11} & 0\\ 0 & 0 & C_{66} \end{array}\right)$$

where two out of its nine elements are independent elastic constants, namely *C*_11_ and *C*_12_. This gives rise to the shear modulus G­(θ), calculated as

3 $$G\left(\theta \right)=C_{66}=\frac{1}{2}\left(C_{11}-C_{12}\right)$$

and two other parameters useful in characterizing mechanical stability of a structure: (i) the Young’s modulus (*Y*(θ)) and (ii) the Poisson’s ratio (ν­(θ)), defined by eqs 4 and 5, respectively

4 $$Y\left(\theta \right)=\frac{C_{11}C_{22}-C_{12}^{2}}{C_{11}\sin^{4}\theta +C_{22}\cos^{4}\theta -\xi }$$

5 $$\nu \left(\theta \right)=\frac{C_{12}\left(\sin^{4}\theta +\cos^{4}\theta \right)-\chi }{C_{11}\sin^{4}\theta +C_{22}\cos^{4}\theta -\xi }$$

where

6 $$\xi =\left(2C_{12}-\frac{C_{11}C_{22}-C_{12}^{2}}{C_{66}}\right)\sin^{2}\theta \cos^{2}\theta$$

7 $$\chi =\left(C_{11}+C_{22}-\frac{C_{11}C_{22}-C_{12}^{2}}{C_{66}}\right)\sin^{2}\theta \cos^{2}\theta$$

Both functions of the angle θ with respect to the positive *x*-axis are as usual.^41^ Besides calculating the elastic stiffness tensor **C** as in eq 2, VASP also computes the compliance tensor (**S** = **C** ^–1^) as a matrix transformation to directly obtain the strain ϵ of a material given a specific stress σ.^42^

Finally, about Raman and IR analysis, the vibrational properties were considered using off-resonance Raman activity and IR spectrum, determined by the method developed by Porezag and co-workers,^43^ focusing on the phonon vibration modes at Γ. For these calculations, we implemented the computational approach proposed by Fonari and Stauffer.^44^ IR and Raman spectra are obtained using a Gaussian smearing of 1 cm^–1^.

## 3 Results and Discussion

### 3.1 Structural and Mechanical Stability

The 2H–MoO_2_ monolayer adopts a hexagonal-lattice structure, with a unit cell containing two O and one Mo atoms in a honeycomb-like arrangement, similar to graphene and other Mo-based TMDCs,^47,48^ as seen in Figure 1 The optimized lattice constant is *a*_0_ = 2.823 Å, with Mo – – –O bond length of 2.039 Å and O – – O separation of 2.451 Å. The Phonon dispersion, shown in Figure 2a, exhibits no imaginary frequencies, confirming dynamical stability. Additionally, the thermodynamic analysis (panel (b) from Figure 2) shows negative Helmholtz free energy above 580 K, suggesting promissing synthesis conditions from temperatures. The constant-volume heat capacity nears the Dulong–Petit limit at 800 K, and entropy increases quasi-linearly up to 500 K.

**Figure 2:**
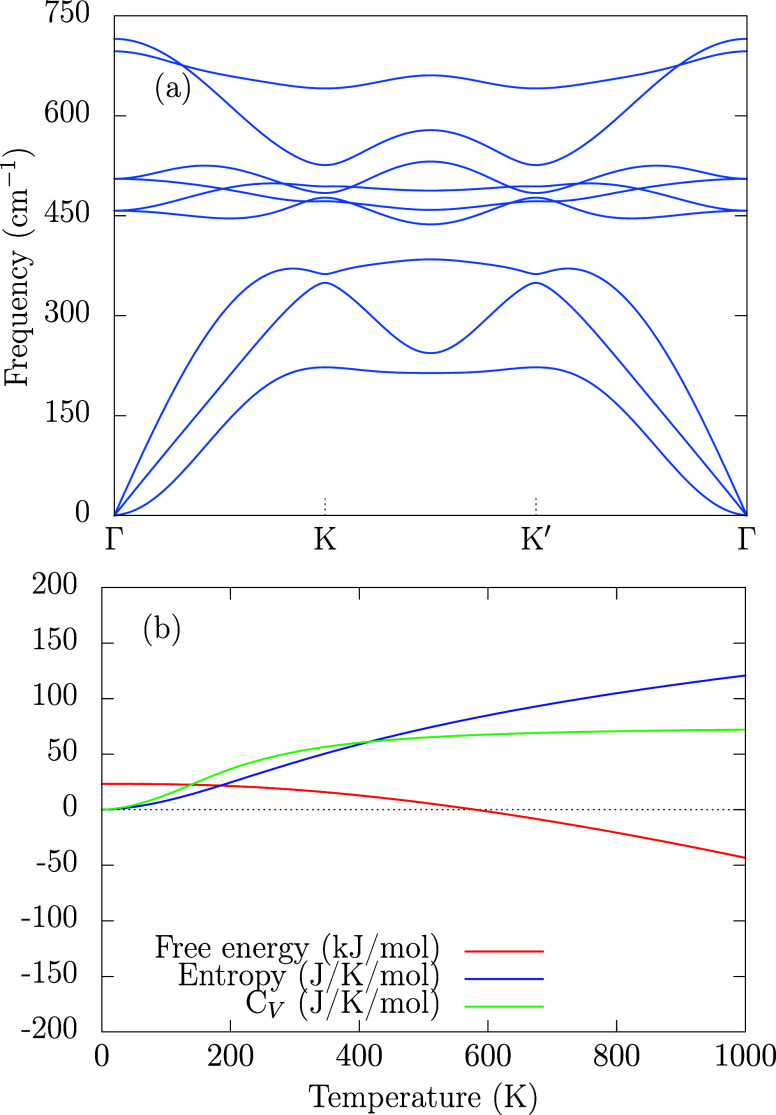
Phonon dispersion and thermodynamic properties of the 2H–MoO_2_ monolayer: (a) phonon band structure and (b) Helmholtz free energy (red), entropy (blue), and constant-volume heat capacity (green).

Our investigated MoO_2_ monolayer presents independent elastic constants of *C*_11_ = 238.8 N/m and *C*_12_ = 85.4 N/m, which satisfy Born’s mechanical stability criteria (*C*_11_ > 0 and *C*_11_ > |*C*_12_|). The derived isotropic moduli include a shear modulus of *G* = 76.2 N/m, a Young’s modulus of *Y* = 207.1 N/m, and a Poisson’s ratio of ν = 0.36. For graphene, the corresponding values are *C*_11_ = 351.4 N/m,^49^ *C*_12_ = 61.6 N/m,^49^ *Y* = 340 N/m,^50^ *G* = 150 N/m,^51^ and ν = 0.398.^52^

When comparing both systems, we find that MoO_2_ exhibits approximately 61% of graphene’s in-plane stiffness (Young’s modulus) and 51% of its shear rigidity, while maintaining a similar Poisson’s ratio, indicating comparable transverse deformation behavior under tensile loading. This lower stiffness suggests that MoO_2_ is mechanically softer and more flexible, potentially facilitating strain engineering and mechanical tunability in devices, whereas graphene remains stiffer and more brittle. Therefore, although MoO_2_ cannot match graphene’s exceptional strength, its moderate elastic constants and good mechanical stability make it a promising material for flexible and deformable electronic or optoelectronic applications. Polar plots, shown in Figure 3, confirm isotropic behavior. Furthermore, ensuring the 2H–MoO_2_ thermodynamic stability, the AIMD thermalization simulation at 300 K shows structural integrity maintained over 5 ps, with energy fluctuations of about 0.07  eV/atom, according to Figure 4a,b.

**Figure 3:**
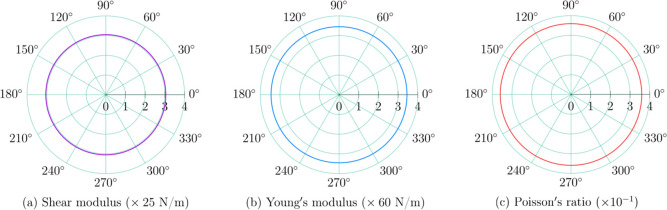
Polar plots of (a) shear modulus *G*(θ), (b) Young’s modulus *Y*(θ), and (c) Poisson’s ratio ν­(θ) for the 2H–MoO_2_ monolayer.

**Figure 4:**
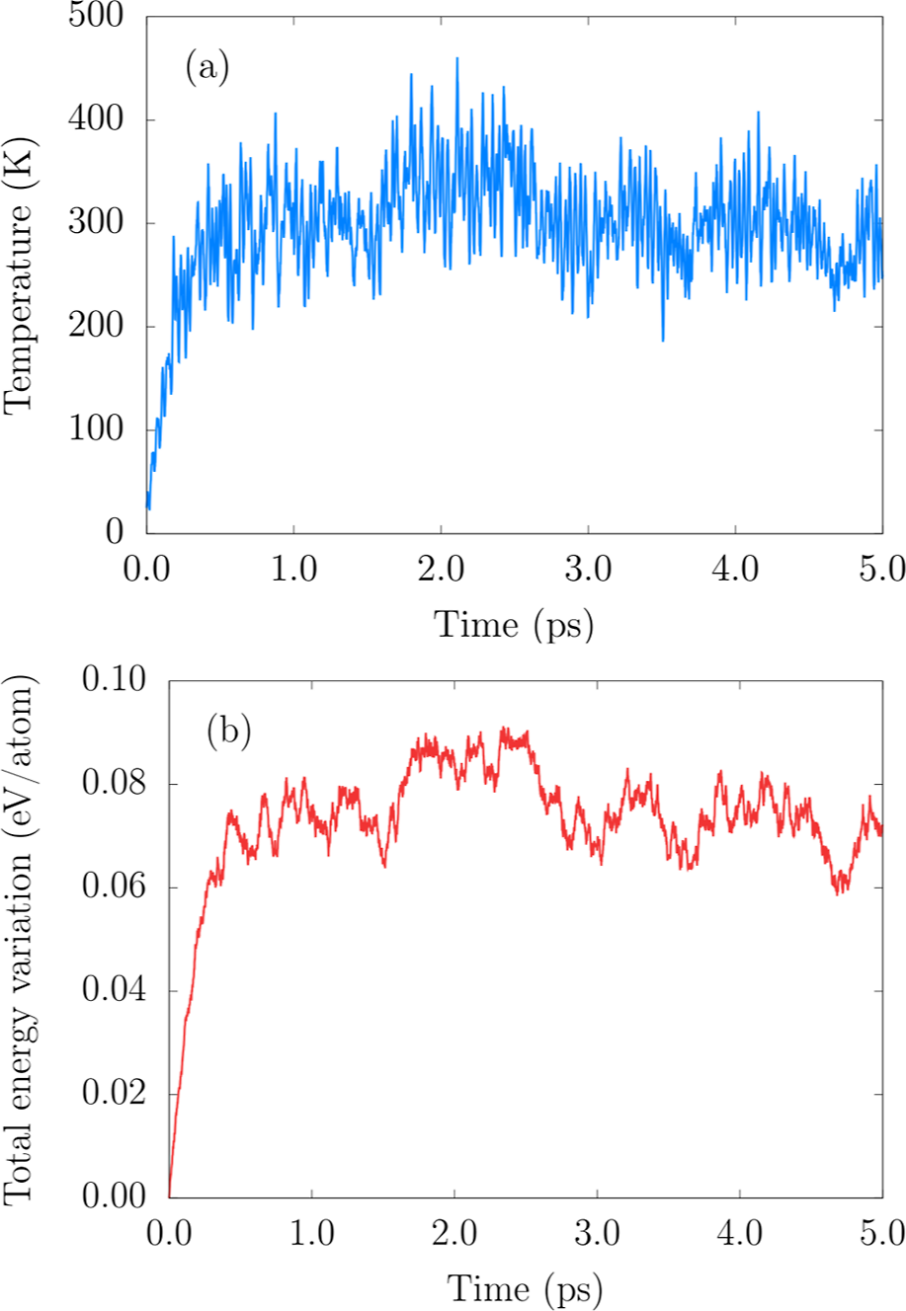
AIMD simulation (thermalization) of the 2H–MoO_2_ monolayer (48-atom supercell): (a) temperature versus time and (b) total energy variation versus time at 300 K.

### 3.2 Electronic Properties

Figure 5 shows that the projected DOS near the Fermi level (0 eV) is dominated by Mo-*d* and O-p orbitals, consistent with semiconducting character. The main contributions for these orbitals occur around −1.80 eV, 1.70 eV, 2.10 eV, and 2.30 eV. In contrast, O-*s* and Mo-p orbitals contribute minimally and appear only as minor features at – 2.0 eV to −1.0 and 2.0 eV to 3.0 eV.

**Figure 5:**
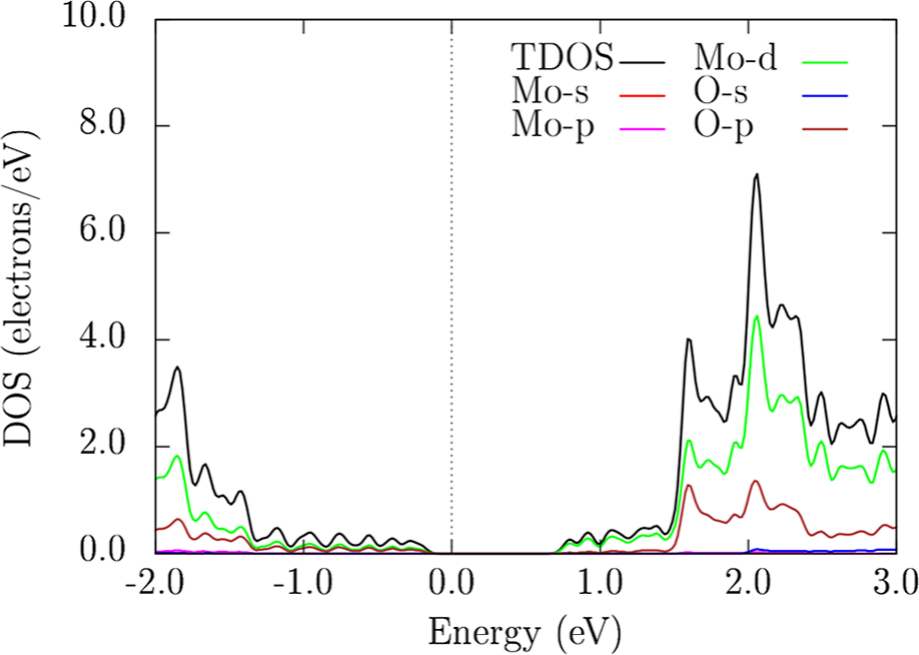
Total and orbital-projected density of states (DOS) of the 2H–MoO_2_ monolayer at the PBE level. The Fermi level is set on 0 eV.

From PBE (blue curves) and PBE + SOC (red curves) band structures, shown in Figure 6a, we observe the PBE prediction of an indirect band gap of 0.93 eV, with the VBM at Γ and the CBM at K/K′, while a direct band gap of 1.76 eV is obtained at Γ. According to the PBE + SOC calculation protocol, our results indicate a negligible SOC effect, with an indirect band gap of 0.92 eV and a direct band gap of 1.70 eV. Notably, spin degeneracy breaks midway between Γ–K and Γ–K′, contrasting with Mo-based TMDCs where splitting occurs at the valleys (making the K and K′ valleys energetically equivalent but with opposite spin configurations).^53,54^

**Figure 6:**
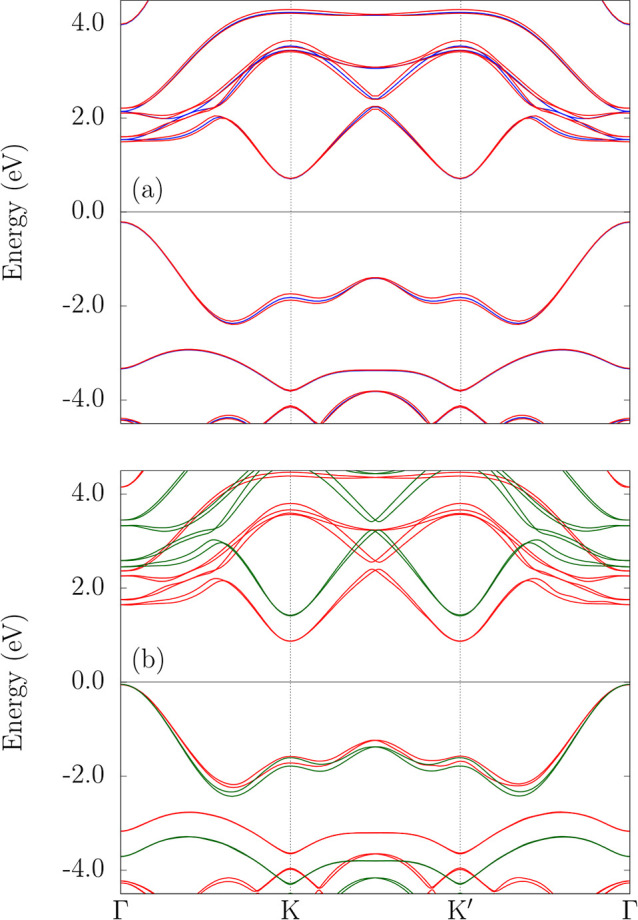
Electronic band structure of the 2H–MoO_2_ monolayer along the Γ–K–K′–Γ path at different theory levels: (a) PBE (blue) and PBE + SOC (red) and (b) PBE + SOC (red) compared with HSE06 + SOC (green).

Comparing the PBE + SOC (red curves) and HSE06+SOC (green curves) band structures in Figure 6 b, we observe that the underestimation of the band gap by PBE becomes evident. While PBE predicts a fundamental gap of 0.92 eV and a direct gap of 1.70 eV, HSE06 corrects these values to 1.55 and 2.50 eV, respectively. This correction not only improves agreement with expected band gap magnitudes but also enhances the SOC-induced spin-degeneracy splitting, in line with the behavior reported for other Mo-based TMDC monolayers.^5^

### 3.3 Raman and IR Spectrum

In the 2H phase, MoO_2_ belongs to the hexagonal crystal system (space group *P*6*m*2) and to the *D*_3*h*_ point group, the same as for monolayer MoS_2_.^55^ The 2H–MoO_2_ primitive cell contains three atoms (two O and one Mo; see Figure 1), yielding nine phonon modes (3*N* = 9 vibrational modes, where *N* is the number of atoms inside the cell): three acoustic and six optical modes, as shown in Figure 7. Among them, the three acoustic modes, located at the **Γ** point, are not active in IR or Raman because they do not produce significant changes in dipole moment or polarizability (see Figure 2a). The remaining six modes are optical and account for the observed IR and Raman peaks.

**Figure 7:**
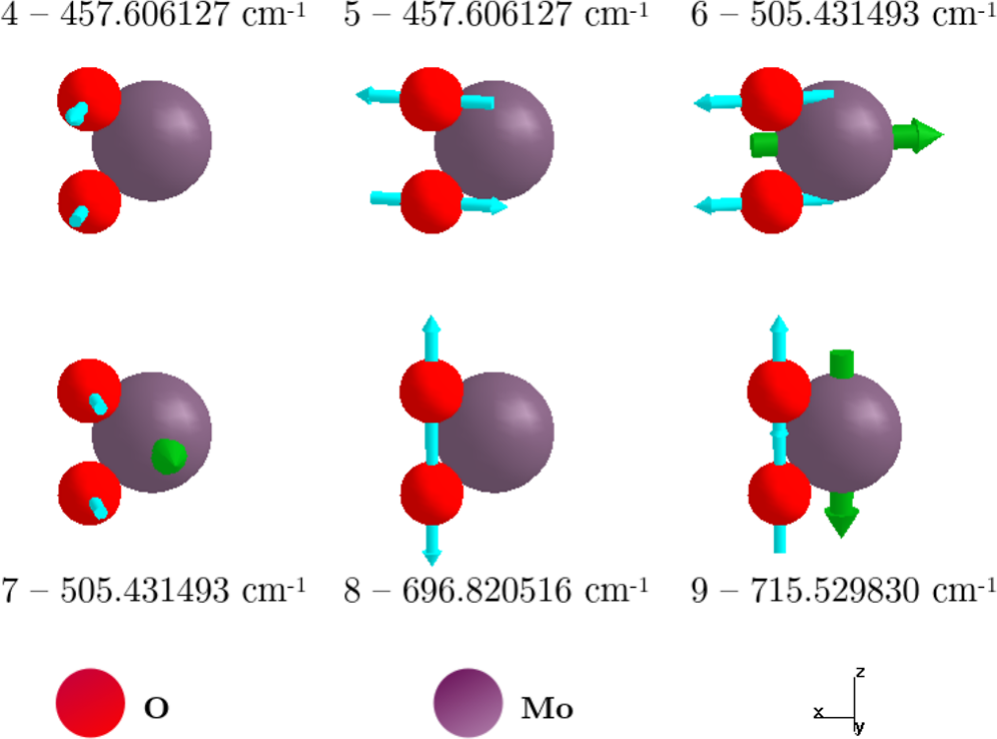
Optical vibrational modes of the 2H–MoO_2_ monolayer at the Γ point.

Figure 8 presents the IR spectrum (a), with a magnification of the 690–705 cm^–1^ region in panel (b), and the Raman spectrum (c), with the same magnified region shown in panel (d), for the 2H–MoO_2_ monolayer. In the IR spectrum (panel (a)), peaks appear at 505 cm^–1^ and 715 cm^–1^, associated with the doubly degenerate *E*_*g*_ mode and the antisymmetric in-plane *A*_*u*_ mode, respectively. In the Raman spectrum (panel (c)), peaks are found at 505 cm^–1^ (*E*_*g*_) and 696 cm^–1^ (*A*_*g*_, out-of-plane stretching). Panels (b) and (d) show magnified views of these *A*_*g*_ and *A*_*u*_ modes, marked with red asterisks; their intensities were scaled for clarity due to their intrinsically weak signals. These assignments agree with symmetry predictions and earlier studies.^55^

**Figure 8:**
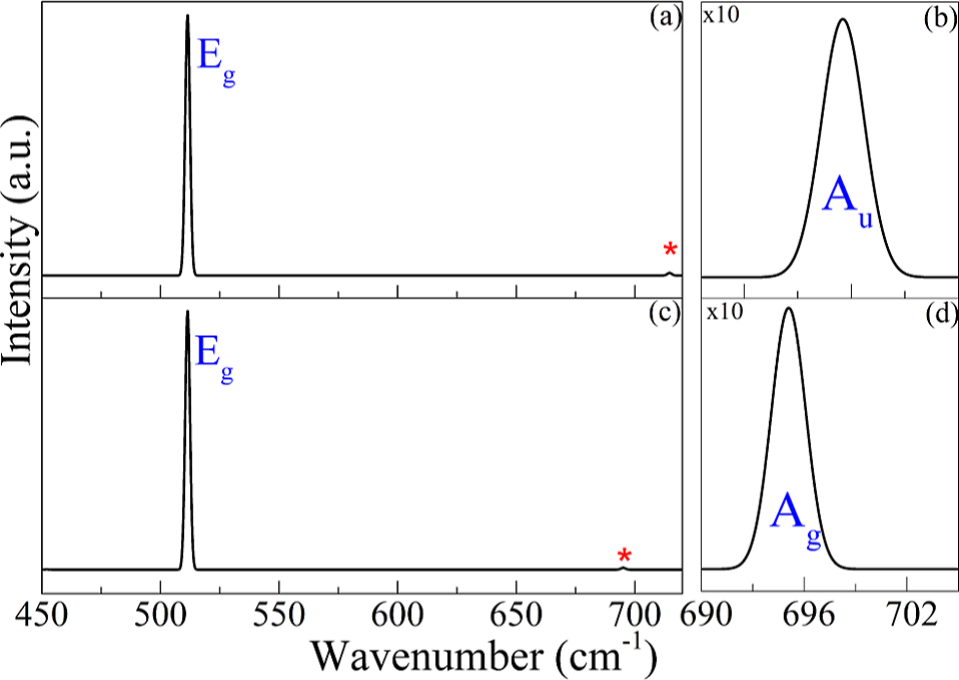
(a) Infrared spectrum and (c) Raman spectrum of the 2H–MoO_2_ monolayer. Panels (b) and (d) show magnified views of the 690–705 cm^–1^ region in the IR and Raman spectra, respectively.

A silent mode is predicted at 457 cm^–1^, which, although symmetry-allowed, is inactive in both IR and Raman spectra because its intensity is below the detection threshold. These vibrational features are also consistent with the phonon dispersion (Figure 2a), where six optical branches emerge from the **Γ** point. Overall, the calculated vibrational modes of 2H–MoO_2_ reproduce the Raman peak positions reported by Ersan et al.,^55^ providing a solid basis for future experimental studies, as this system has not yet been extensively explored.

### 3.4 Excitonic and Optical Properties

We investigated the excitonic properties of the 2H–MoO_2_ monolayer by calculating the excitonic band structure shown in Figure 9. Four local minima are observed in the vicinity of the K and K′ valleys, with an indirect exciton ground state of 1.19 eV located at the K valley, and a direct exciton state at 2.11 eV. The resulting exciton binding energy, defined as the difference between the HSE06 + SOC fundamental band gap and the indirect exciton ground state, is 0.38 eV. This value is consistent with those typically reported for 2D materials.^5,56–58^

**Figure 9:**
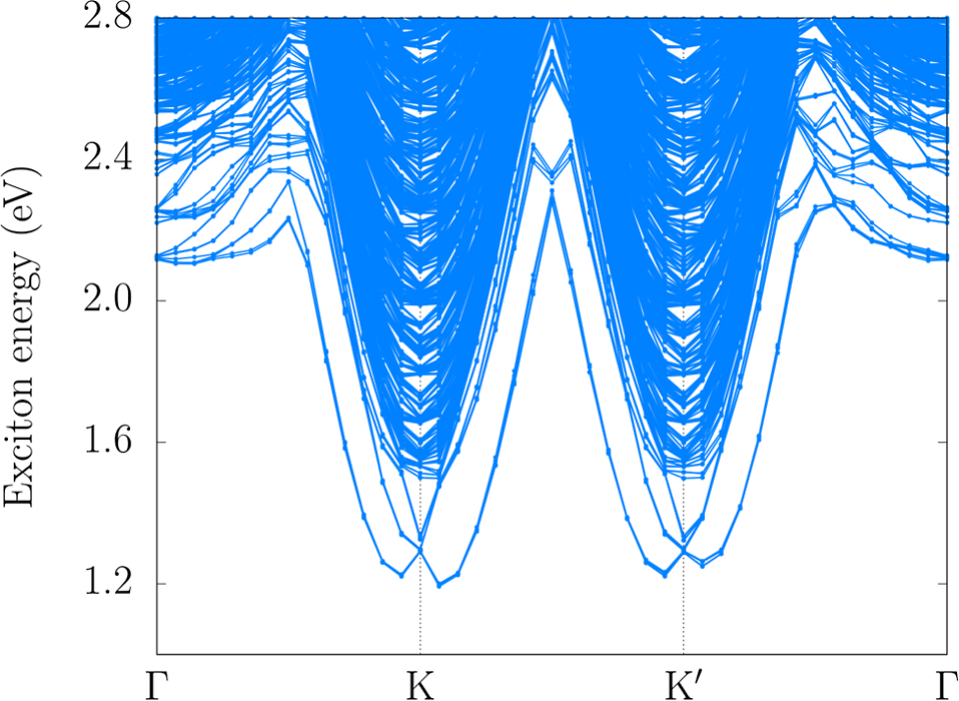
MoO_2_ monolayer exciton band structure along the **k**-path **Γ**–K–K′–**Γ**.

The linear optical response–obtained at both the IPA and BSE levels–is shown in Figure 10 for the absorption coefficient (a), refractive index (b), and reflectivity (c). Our calculations were performed for linear light polarization along the x̂ and ŷ directions. The spectra present only small variations with polarization, regardless of the level of theory, establishing the isotropic optical behavior of the 2H–MoO_2_ monolayer. Specifically, panel (a) reveals that excitonic effects strongly influence the absorption onset, red-shifting the optical gap from 2.50 eV (IPA) to 2.11 eV (BSE). At the BSE level, absorption starts at 2.11 eV and exhibits repeated peaks from 2.49 to 4.00 eV, reaching a maximum of 4.61 × 10^5^ cm^–1^ at 2.64 eV. Excitonic effects, therefore, enhance the absorption intensity across the visible and ultraviolet ranges, with a marginal preference for the x̂ polarization in the visible.

**Figure 10:**
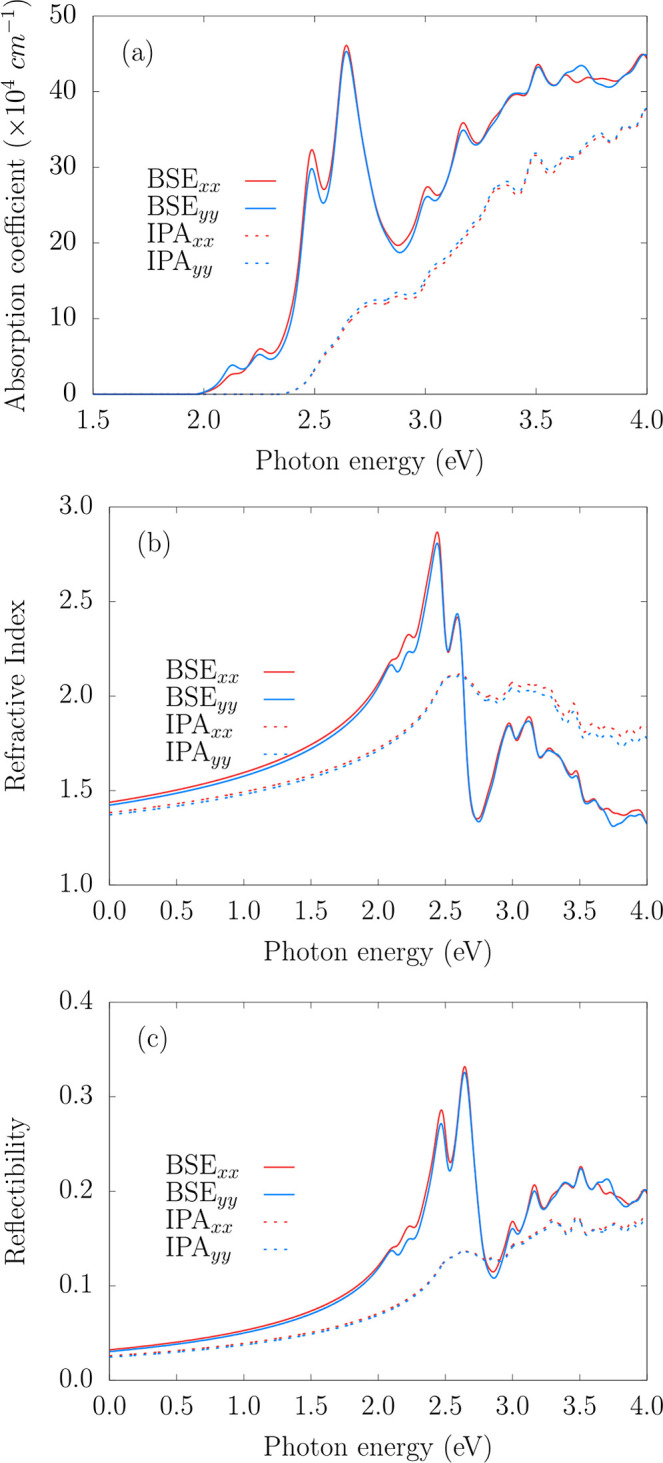
Optical properties of the MoO_2_ monolayer: (a) absorption coefficient, (b) refractive index, and (c) reflectivity at BSE (solid lines) and IPA (dashed lines) levels for linear light polarization along the *x*-(red) and *y*-(blue) directions.

The refractive index and reflectivity, as depicted in Figure 10b,c, display similar trends. The refractive index increases steadily in the IR region, reaching 2.87 at 2.44 eV, before sharply decreasing to 1.34 at 2.75 eV. Reflectivity exhibits peaks of 0.29 and 0.33 at 2.47 and 2.64 eV, respectively, and a minimum of 0.11 at 2.86 eV. Excitonic effects dominate the refractive index up to 2.63 eV, beyond which IPA values become slightly larger. For reflectivity, excitonic contributions prevail over most of the energy range, except for a narrow visible interval between 2.80 and 2.91 eV, where IPA values exceed those from BSE.

## 4 Conclusion

In this work, we carried out a comprehensive characterization of the 2H–MoO_2_ monolayer, focusing on its structural, electronic, optical, and excitonic properties using first-principles calculations based on DFT. This TMDO adopts a hexagonal lattice with an equilibrium lattice constant of 2.823 Å and exhibits structural stability, indicating a favorable synthesis above 580 K. Such stability was confirmed by phonon dispersion, which displayed no imaginary frequencies. AIMD simulations at 300 K (thermalization) revealed energy fluctuations of approximately 0.07  eV/atom over the simulation period, further supporting the structural robustness of the system. Mechanical stability was verified by elastic constants *C*_11_ and *C*_12_, which satisfy the Born criteria. Additionally, the isotropic character of the structure was highlighted by symmetric plots of Poisson’s ratio, shear modulus, and Young’s modulus. Electronic analysis revealed that O-p and Mo-d orbitals dominate the DOS near the Fermi level, confirming semiconducting behavior. The material exhibits a direct band gap of 2.50 eV at the HSE06+SOC level, with SOC-induced splitting evident along most of the high-symmetry **k**-path, except near the Γ point for the top valence band. Vibrational analysis identified strong IR and Raman activity at 505 cm^–1^ (*E*_*g*_ mode), with additional features at 715 cm^–1^ (*A*_*u*_, IR) and 696 cm^–1^ (*A*_*g*_, Raman). Excitonic calculations yielded a direct exciton ground state at 2.12 eV and a binding energy of 0.38 eV, values consistent with those of similar 2D monolayers. Optical spectra computed at both the IPA and BSE levels showed significant excitonic effects, notably a redshift of the absorption edge. Nonetheless, the absorption coefficient, refractive index, and reflectivity curves displayed minimal polarization dependence, confirming the optical isotropy of the system. Thus, the 2H–MoO_2_ monolayer emerges as a structurally stable and optically isotropic semiconductor with strong excitonic effects and broadband absorption across the visible and ultraviolet regions. Together, these properties make it a promising candidate for applications in optoelectronic and photovoltaic devices requiring consistent light absorption, as well as in optical components designed to operate across a wide frequency range.
